# The oncoprotein HBXIP promotes glucose metabolism reprogramming via downregulating SCO2 and PDHA1 in breast cancer

**DOI:** 10.18632/oncotarget.4508

**Published:** 2015-07-30

**Authors:** Fabao Liu, Weiying Zhang, Xiaona You, Yunxia Liu, Yinghui Li, Zhen Wang, Yue Wang, Xiaodong Zhang, Lihong Ye

**Affiliations:** ^1^ State Key Laboratory of Medicinal Chemical Biology, Department of Biochemistry, College of Life Sciences, Nankai University, Tianjin, P.R. China; ^2^ State Key Laboratory of Medicinal Chemical Biology, Department of Cancer Research, College of Life Sciences, Nankai University, Tianjin, P.R. China

**Keywords:** HBXIP, SCO2, PDHA1, glucose metabolism reprogramming, breast cancer

## Abstract

The glucose metabolism reprogramming is a hallmark of cancer. The oncoprotein hepatitis B X-interacting protein (HBXIP) functions in the development of breast cancer. In this study, we supposed that HBXIP might be involved in the glucose metabolism reprogramming in breast cancer. We showed that HBXIP led to increases in generation of intracellular glucose and lactate, as well as decreases in generation of reactive oxygen species. Expression of synthesis of cytochrome c oxidase 2 (SCO2) and pyruvate dehydrogenase alpha 1 (PDHA1), two factors of metabolic switch from oxidative phosphorylation to aerobic glycolysis, was suppressed by HBXIP. In addition, miR-183/182 and miR-96 directly inhibited the expression of SCO2 and PDHA1 through targeting their mRNA coding sequences (CDSs), respectively. Interestingly, HBXIP elevated the miR-183/96/182 cluster expression through hypoxia-inducible factor 1α (HIF1α). The stability of HIF1α was enhanced by HBXIP through disassociating interaction of *von Hippel-Lindau* protein (pVHL) with HIF1α. Moreover, miR-183 increased the levels of HIF1α protein through directly targeting CDS of VHL mRNA, forming a feedback loop of HIF1α/miR-183/pVHL/HIF1α. In function, HBXIP-elevated miR-183/96/182 cluster enhanced the glucose metabolism reprogramming *in vitro*. HBXIP-triggered glucose metabolism reprogramming promoted the growth of breast cancer *in vivo*. Thus, we conclude that the oncoprotein HBXIP enhances glucose metabolism reprogramming through suppressing SCO2 and PDHA1 in breast cancer.

## INTRODUCTION

Most cancer cells rely on aerobic glycolysis to generate the energy needed by cellular processes, which is termed the “Warburg effect” [1–3]. Much of the metabolic variation among individuals is genetically defined, the mutations in oncogenes (e.g., c-Myc and HIF1) and tumor suppressor genes (e.g., p53 and PTEN), which tend to enhance aerobic glycolysis and energy production, may represent a well-recognized mechanism [2, 4]. Human synthesis of cytochrome c oxidase 2 (SCO2), a novel p53-inducible protein, is a cytochrome c oxidase (COX) assembly protein that participates in the energy-generating metabolic pathways mediated by p53 through modulating the balance between the utilization of respiratory and glycolytic pathways [2, 5–9]. Disruption of SCO2 in human cancer cells with wild-type p53 triggers the metabolic switch from oxidative phosphorylation (OXPHOS) to glycolysis [6, 10]. Severe COX deficiency is observed in patients and mouse models with loss of function of SCO2 [11, 12]. SCO2 functions as an apoptotic protein in xenograft tumors through increasing the generation of reactive oxygen species (ROS) and inducing dissociation of the ASK-1-Trx complex [5]. Pyruvate dehydrogenase (PDH), a highly regulated and less active enzyme, provides the link between glycolysis and the tricarboxylic acid (TCA) cycle [4, 13]. PDH E1 alpha (PDHA1) is a pivotal and rate-limiting E1α subunit for the PDH complex [14]. PDHA1 knockdown results in enhanced glucose uptake, rate of glycolysis and lactate production in H460-con cells [15].

Hypoxia-inducible factor α (HIF1α) is one of the transcriptional factors frequently activated in the tumors and widely involved in the tumor growth, progression, and resistance to chemotherapy. In fact, HIF1α with NF-κB can regulate the transcription of more than one thousand of genes that, in turn, controls vital cellular processes such as adaptation to the hypoxia, metabolic reprogramming, or malignant progression [16]. Despite HIF1α is unstable in well-oxygenated tissues owing to ubiquitin-mediated degradation, additional factors have been identified that can cause HIF1α accumulation, and also regulate metabolism in aerobic conditions [17, 18]. In cancers, oncogenic mutations and accumulation of intermediate metabolites contribute to the HIF1 regulation in better-oxygenated conditions [17]. pVHL, as the product of *Von Hippel-Lindau* (VHL) tumor suppressor gene, functions as the substrate recognition component of an E3-ubiquitin ligase complex marking specific target proteins for degradation. Historically, pVHL was reported to interact with HIF1α [19–23]. pVHL directs the polyubiquitylation of HIF1α to degrade in the proteasome.

Mammalian hepatitis B X-interacting protein (HBXIP) is originally identified for its interaction with the C terminus of the hepatitis B virus X protein [24]. However, many studies show that HBXIP acts as an oncoprotein in controlling cell proliferation, apoptosis and division [25, 26]. HBXIP serves as a regulator component required for mTORC1 activation by amino acids [27]. Our group shows that HBXIP imports into the nucleus of breast cancer cells, acting as a transcriptional coactivator, to promote the progression of breast cancer [28–32]. HBXIP is able to upregulate the HIF1α expression through FGF8/PI3K/Akt signaling in breast cancer [33]. However, it remains poorly understood whether HBXIP is involved in the glucose metabolism reprogramming in breast cancer.

In this study, we are interested in the effect of HBXIP on the glucose metabolism reprogramming in breast cancer. Our data show that HBXIP promotes the glucose metabolism reprogramming through downregulating SCO2 and PDHA1 in breast cancer cells, involving HIF1α/miR-183/96/182 cluster/pVHL signaling. Our finding provides new insights into the mechanism by which HBXIP enhances the glucose metabolism reprogramming in breast cancer.

## RESULTS

### HBXIP regulates glucose metabolism reprogramming and downregulates SCO2 and PDHA1 in breast cancer

**Figure 1:**
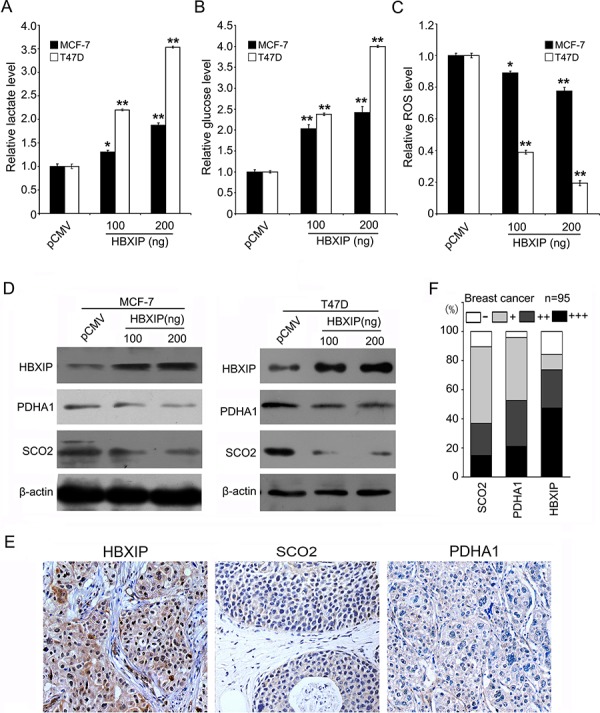
HBXIP regulates glucose metabolism reprogramming and downregulates SCO2 and PDHA1 in breast cancer **A.** The levels of lactate in the culture media of MCF-7 and T47D cells were measured by an Agilent 1100 series high-performance liquid chromatography (HPLC) system and normalized to cell number. **B.** The levels of intracellular glucose were detected by glucose-lactate biosense tester SBA-40E and normalized based on the protein concentration in MCF-7 and T47D cells. **C.** The levels of intracellular ROS were assessed by flow cytometry analysis in MCF-7 and T47D cells. **D.** The protein levels of HBXIP, PDHA1 and SCO2 were examined by Western blot analysis in MCF-7 and T47D cells. **E.** The expression levels of SCO2, PDHA1 and HBXIP protein were examined by IHC analysis in clinical breast cancer tissues using tissue microarrays, which were from the same tissue paraffin block. **F.** The percentage of staining gradations of SCO2, PDHA1 and HBXIP of tissue microarrays containing 95 cases of clinical breast cancer tissues was shown. Statistically significant differences are indicated: *P < 0.05, **P < 0.01, Student's *t*-test. Each experiment was repeated at least three times. Data are shown as mean ± SEM (*n* = 3).

Growing evidence suggests that HBXIP abundance is increased in breast cancer tissues and markedly accelerates breast cancer growth [28–31]. In this study, we are interested in whether HBXIP is involved in the glucose metabolism reprogramming of breast cancer. Interestingly, we uncovered that HBXIP significantly increased generation of lactate and intracellular glucose and then reduced intracellular ROS in MCF-7 and T47D cells (Figure 1A–1C), suggesting that HBXIP might be associated with the glucose metabolism reprogramming. The p53 was reported to inhibit glycolysis and increase OXPHOS [6, 7]. We concerned whether HBXIP enhanced the glucose metabolism reprogramming in a p53-dependent manner. Then, we compared the differences of lactate, intracellular glucose and ROS between the wild-type p53 expressing MCF-7 cells and mutant p53 (L194F) expressing T47D cells when the cells were treated with si-p53 or si-p53/si-HBXIP. As expected, p53 knockdown obviously increased generation of lactate, intracellular glucose and decreased ROS. Interestingly, the treatment with si-p53/si-HBXIP resulted in inhibition of the event in both MCF-7 and T47D cells (Supplementary Figure 1A), meanwhile, the interference efficiency of si-p53 and si-HBXIP was confirmed by Western blot analysis, suggesting that HBXIP affects the glucose metabolism reprogramming in a p53-independent manner. SCO2 and PDHA1 were closely correlated to the metabolic switch from OXPHOS to glycolysis [6, 7]. Moreover, we found that HBXIP could significantly reduce the levels of SCO2 and PDHA1 protein in MCF-7 and T47D cells in a dose-dependent manner, but little effect was observed at the mRNA level (Figure 1D; Supplementary Figure 1B). The positive staining of SCO2, PDHA1 and HBXIP in clinical breast cancer samples was showed in Figure 1E and 1F. Interestingly, the positive rates of SCO2 and PDHA1 were 25.7% (18/70) and 41.4% (29/70) in HBXIP-strong positive tissues (74.0%, 70/95). Overall, we conclude that HBXIP regulates the glucose metabolism reprogramming and downregulates SCO2 and PDHA1 in breast cancer.

### MiR-183/96/182 cluster downregulates SCO2 and PDHA1 through targeting their mRNA CDSs

**Figure 2:**
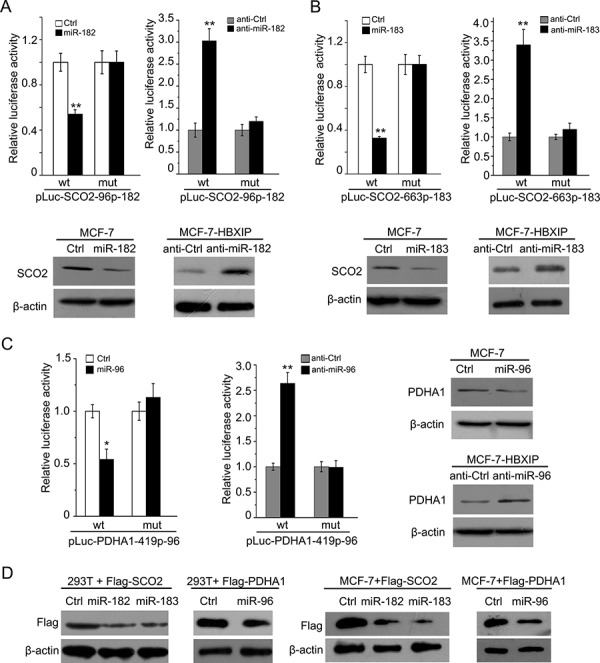
MiR-183/96/182 cluster downregulates SCO2 and PDHA1 through targeting their mRNA CDSs **A, B, C.** The relative luciferase activities of wild-type (wt) and mutant (mut) pLuc-SCO2–96p-182 (pLuc-SCO2–663p-183) or pLuc-PDHA1-419p-96 were detected by luciferase reporter gene assays in MCF-7 cells, followed by Western blot analysis. **D.** The ectopic expression of Flag-SCO2 or Flag-PDHA1 was detected by Western blot analysis in 293T and MCF-7 cells. The Ctrl and anti-Ctrl were used as controls of miRNA and its inhibitor. Statistically significant differences are indicated: *P < 0.05, **P < 0.01, Student's *t*-test. Each experiment was repeated at least three times. Data are shown as mean ± SEM (*n* = 3).

Next, we try to identify the mechanism by which HBXIP downregulates SCO2 and PDHA1 in breast cancer cells. We used the computational approach RNA22 to determine the miRNAs that target the 3′ untranslated region (3′UTR) and coding sequences (CDSs) of SCO2 and PDHA1 mRNAs [34, 35]. We predicted that miR-183 and miR-182 might target SCO2 mRNA CDS, and miR-96 might target PDHA1 mRNA CDS (Supplementary Figure 2A and 2B). It has been reported that the productions of miR-183/96/182 cluster function as oncogenes involving in tumor growth and progression [36, 37]. To validate the effect of miR-183 (miR-182) or miR-96 on the expression of SCO2 or PDHA1, we cloned the miRNA recognition element (MRE) and its mutant into pGL3-Control plasmid, respectively. Our data showed that the luciferase activities of the MRE of SCO2–96p-182-wt (SCO2–663p-183-wt or PDHA1-419p-96-wt) could be significantly suppressed by its matching miRNA, but it failed to work when the above MREs were mutated. The luciferase activities of above MREs were increased by the inhibitors of corresponding miRNAs, which could be attenuated when the target sites were mutated. Meanwhile, each miRNA could decrease the protein levels of its target gene in MCF-7 cells and each miRNA inhibitor resulted in the opposite data in MCF-7-HBXIP cells (Figure 2A–2C). In addition, we validated the expression levels of miR-183, miR-96 and miR-182 by qRT-PCR analysis (Wilcoxon signed-rank test, Supplementary Figure 2C), and HBXIP expression by IHC analysis in clinical breast cancer tissues (Supplementary Figure 2D). To further confirm these observations, we constructed the full-length SCO2 and PDHA1 in a Flag-tagged vector and cotransfected them into 293T and MCF-7 cells with their matching miRNAs. As expected, Western blot analysis showed that the exogenous expression of SCO2 and PDHA1 could be decreased by miRNAs in the cells (Figure 2D), suggesting that miR-183/miR-182 are able to target mRNA CDS of SCO2 and miR-96 is able to target mRNA CDS of PDHA1. Therefore, we conclude that miR-183/96/182 cluster is able to downregulate the expression of SCO2 and PDHA1 at the post-transcriptional level in breast cancer cells.

### HBXIP activates miR-183/96/182 promoter through transcriptional factor HIF1α

**Figure 3:**
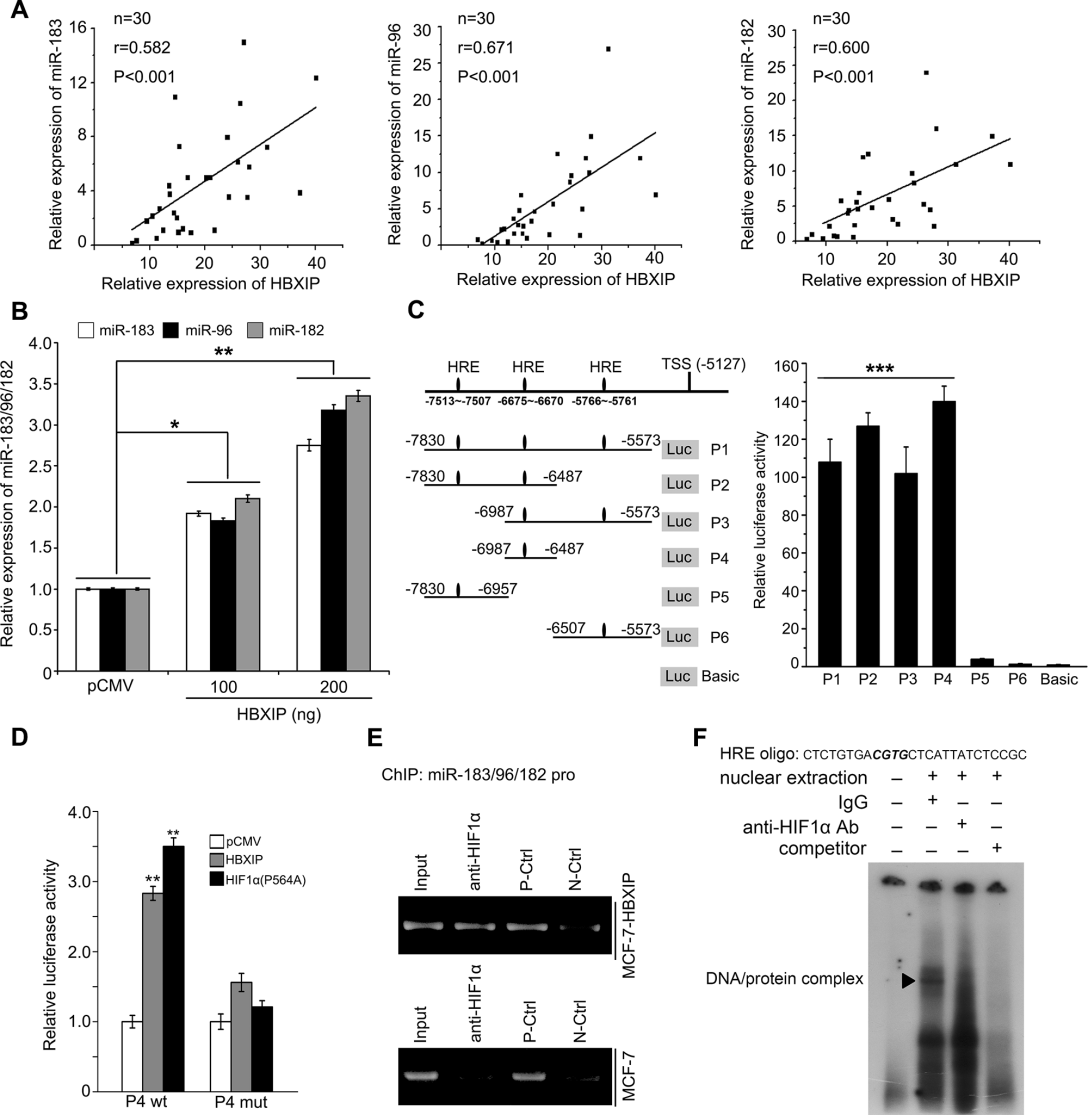
HBXIP activates miR-183/96/182 promoter through transcriptional factor HIF1α **A.** The correlations of HBXIP mRNA levels with miR-183 (miR-96 or miR-182) levels were detected by qRT-PCR analysis in 30 cases of clinical breast cancer tissues. **B.** The relative expression levels of miR-183, miR-96 and miR-182 were measured by qRT-PCR analysis in MCF-7 cells. **C.** Schematic diagram shows the miR-183/96/182 promoter with hypoxia response elements (HREs). The promoter activities of miR-183/96/182 cluster were determined by luciferase reporter gene assays in MCF-7 cells. **D.** The promoter activities of P4 (−6987~ −6487) of miR-183/96/182 were measured by luciferase reporter gene assays in MCF-7 cells. **E.** The interaction of HIF1α with the promoter region of miR-183/96/182 cluster was examined by ChIP assays in MCF-7 and MCF-7-HBXIP cells. **F.** The interaction of HIF1α with HRE located in the region of miR-183/96/182 promoter was examined by EMSA in MCF-7 cells. Statistically significant differences are indicated: *P < 0.05, **P < 0.01, Student's *t*-test. Each experiment was repeated at least three times. Data are shown as mean ± SEM (*n* = 3).

According to above observation that HBXIP downregulates SCO2 and PDHA1 which are targeted by miR-183/96/182, we are interested in whether HBXIP is able to upregulate miR-183/96/182 in breast cancer cells. Our data showed that the expression levels of HBXIP were positively correlated to those of miR-183, miR-96 and miR-182 by qRT-PCR analysis in clinical breast cancer tissues, respectively (Figure 3A). Moreover, we validated that the overexpression of HBXIP was able to upregulate the expression of miR-183, miR-96 and miR-182 in MCF-7 cells, respectively (Figure 3B). To better understand the mechanism by which HBXIP upregulated miR-183/96/182, we analyzed the promoter region of −7830~ −5573 of miR-183/96/182 locus by promoter analysis program TF SEARCH and Genomatix software suite. Interestingly, we found that there were three conserved hypoxia response elements (HREs) located in the region. Our previous study showed that HBXIP was able to upregulate HIF1α expression through FGF8/PI3K/Akt signaling in MCF-7 cells [33]. Therefore, we supposed that HBXIP might activate miR-183/96/182 promoter through transcriptional factor HIF1α. Then, we cloned six constructs of different fragments of miR-183/96/182 locus promoter. Luciferase reporter gene assays indicated that the fragment of P4 (−6987~ −6487) containing one HRE exhibited the maximum luciferase activities (Figure 3C), suggesting that the fragment −6987~ −6487 is the core region of miR-183/96/182 locus promoter and the transcriptional factor HIF1α may contribute to the activation of miR-183/96/182 promoter. Moreover, we revealed that the overexpression of HBXIP was able to activate each fragments containing the HRE (Supplementary Figure 3A), suggesting that HBXIP is able to activate miR-183/96/182 promoter through transcriptional factor HIF1α. To further evaluate whether HIF1α is involved in the activation of miR-183/96/182 promoter mediated by HBXIP, we used cobalt chloride (CoCl_2_), a chemical inducer of HIF1α, to test the effect of HIF1α on promoter activity of miR-183/96/182. Interestingly, luciferase reporter gene assays showed that the promoter activities of P4 were significantly higher relative to control in MCF-7 cells (Supplementary Figure 3B). Moreover, we showed that a prolyl-hydroxylation-defective mutant of HIF1α such as HIF1α (P564A) [38], which escaped the pVHL control, could remarkably elevate the promoter activities of P4 of miR-183/96/182 in the cells, but HBXIP or HIF1α (P564A) failed to work when the HIF1α binding sites were mutated (P4 mut) (Figure 3D). Moreover, ChIP assays revealed that HIF1α was able to bind to the miR-183/96/182 promoter in MCF-7-HBXIP cells (Figure 3E). We further evaluated the presence of HIF1α on the miR-183/96/182 promoter by electrophoretic mobility shift assays (EMSA). Our data showed that antibodies against HIF1α could completely disrupt the DNA/protein complex (Figure 3F), suggesting that HIF1α activates miR-183/96/182 promoter through binding to the promoter. Thus, we conclude that HBXIP upregulates miR-183/96/182 through transcriptional factor HIF1α in breast cancer cells.

### HBXIP increases the protein level of HIF1α through blocking the degradation of HIF1α to activate miR-183/96/182

**Figure 4:**
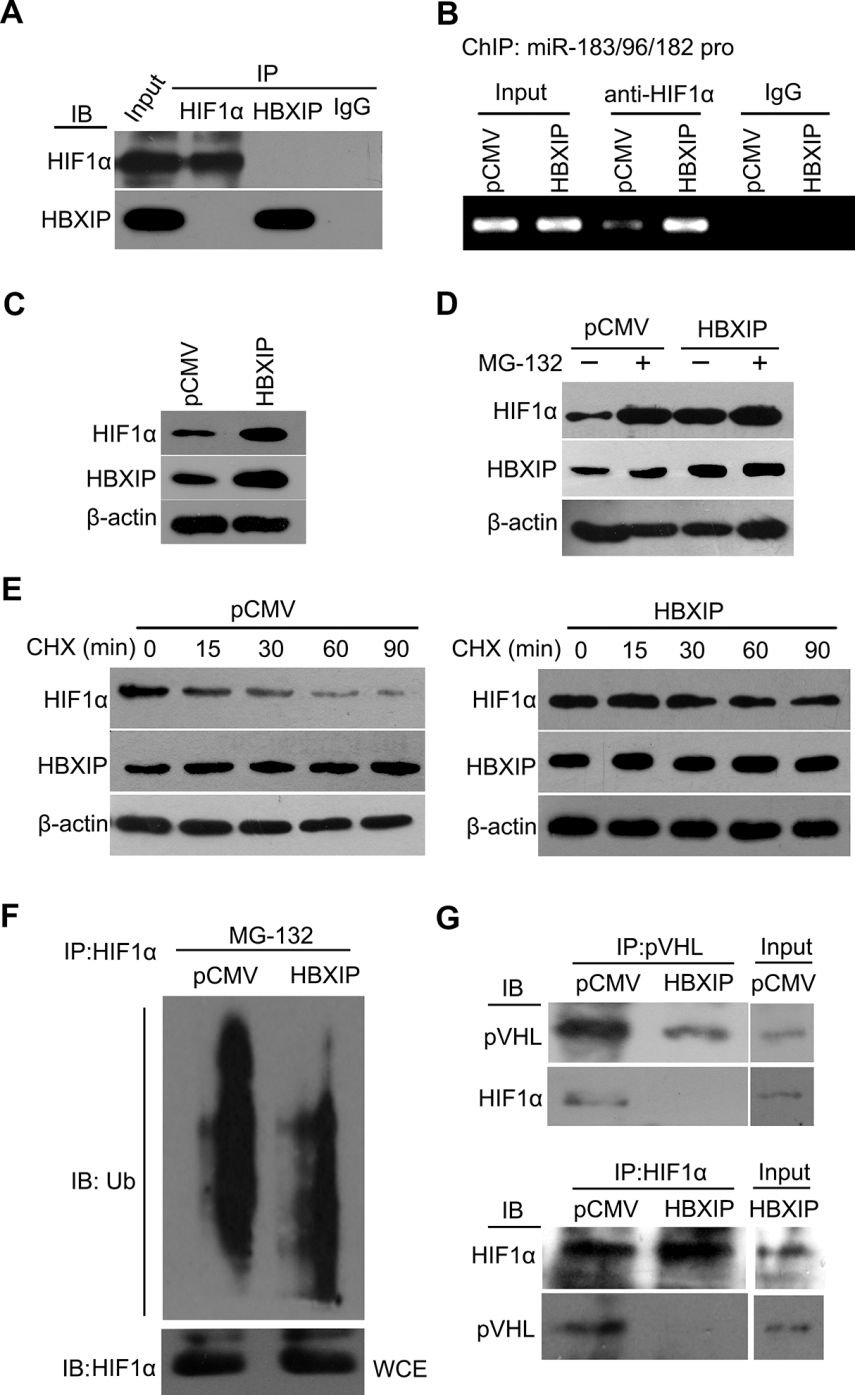
HBXIP increases the protein level of HIF1α through blocking the degradation of HIF1α to activate miR-183/96/182 **A.** The interaction of HIF1α with HBXIP was examined by co-IP assays in 293T cells, followed by Western blot analysis. **B.** The interaction of HIF1α with the promoter region of miR-183/96/182 cluster was examined by ChIP assays in MCF-7 cells. **C.** In ChIP assays, the expression of HBXIP and HIF1α was validated by Western blot analysis. **D.** The expression of HIF1α and HBXIP in MCF-7 cells treated with 25 mM MG-132 was examined by Western blot analysis. **E.** The expression of HIF1α and HBXIP in MCF-7 cells treated with 100 mg/mL cycloheximide (CHX) was examined by Western blot analysis. **F.** The ubiquitination levels of HIF1α were measured by Western blot analysis in MCF-7 cells. **G.** The interaction of HIF1α with pVHL was examined by co-IP assays in MCF-7 cells, followed by Western blot analysis. Each experiment was repeated at least three times.

Next, we examined whether HBXIP activated miR-183/96/182 promoter through interaction with HIF1α in breast cancer cells. Immunoprecipitation assays showed that HBXIP failed to bind to HIF1α in the cells (Figure 4A). However, ChIP assays revealed that HBXIP increased the binding of HIF1α to the promoter of miR-183/96/182 (Figure 4B). Meanwhile, we validated that HBXIP overexpression upregulated HIF1α at the protein level (Figure 4C), but the expression of HIF1α could be downregulated by the knockdown of HBXIP in MCF-7-HBXIP cells (Supplementary Figure 3C), suggesting that HBXIP activates miR-183/96/182 promoter through upregulating transcriptional factor HIF1α, rather than coactivating HIF1α. We previously showed that HBXIP was able to upregulate HIF1α expression through FGF8/PI3K/Akt signaling in MCF-7 cells [33]. HIF1α is unstable in well-oxygenated tissues owing to ubiquitin-mediated degradation [39], we further concerned whether HBXIP affected the stability of HIF1α as well. The treatment with the proteasome inhibitor MG-132 or HBXIP overexpression resulted in accumulation of HIF1α, but HBXIP overexpression failed to affect MG-132-mediated accumulation of HIF1α (Figure 4D). Moreover, the half-life assays showed that HBXIP overexpression could obviously suppress the degradation of HIF1α (Figure 4E), suggesting that HBXIP contributes to the stability of HIF1α in breast cancer cells. Low level of HIF1α protein during normoxia resulted from the promotion of its oxygen-dependent degradation by the pVHL pathway [40]. Accordingly, we further validated that HBXIP reduced ubiquitination of HIF1α through disassociation the interaction of HIF1α with pVHL (Figure 4F and 4G). Thus, we conclude that HBXIP increases the protein level of HIF1α through suppression of degradation of HIF1α to activate miR-183/96/182.

### MiR-183 enhances the stability of HIF1α through targeting VHL mRNA CDS

**Figure 5:**
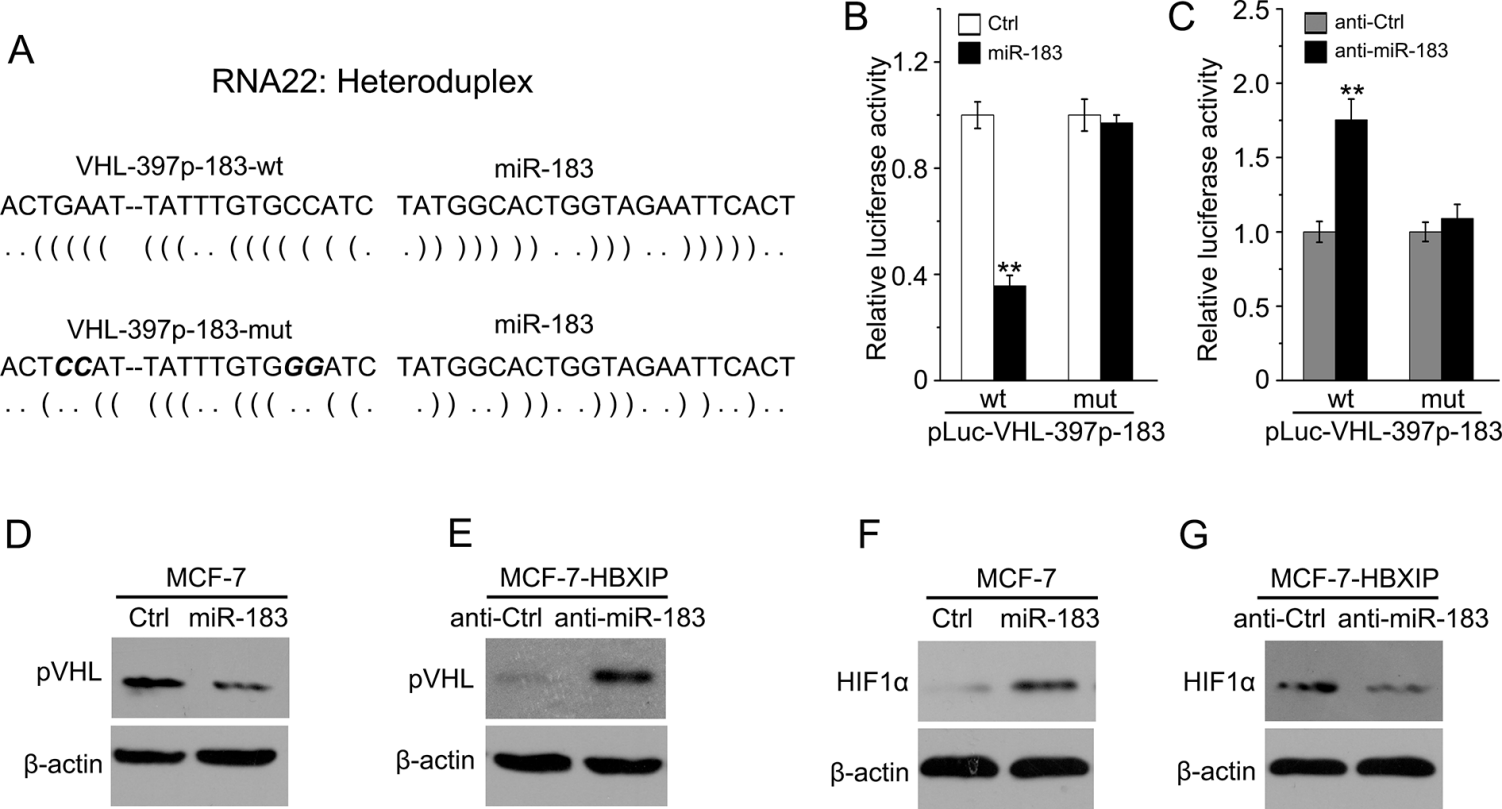
MiR-183 enhances the stability of HIF1α through targeting VHL mRNA CDS **A.** The schematic diagram shows the heteroduplexes of VHL mRNA CDS and its mutant in the miR-183 recognition elements (MREs). **B, C.** The relative luciferase activities of wild-type and mutant pLuc-VHL-397p-183 were detected by luciferase reporter gene assays in MCF-7 cells transfected with miR-183 (or in MCF-7-HBXIP cells transfected with anti-miR-183). **D, E.** The expression levels of pVHL were detected by Western blot analysis in MCF-7 cells transfected with miR-183 (or in MCF-7-HBXIP cells transfected with anti-miR-183). **F, G.** The expression levels of HIF1α were measured by Western blot analysis in MCF-7 cells transfected with miR-183 (or in MCF-7-HBXIP cells transfected with anti-miR-183). Statistically significant differences are indicated: **P < 0.01, Student's *t*-test. Each experiment was repeated at least three times. Data are shown as mean ± SEM (*n* = 3).

Given that interaction of pVHL with HIF1α directed the polyubiquitylation of HIF1α to degrade in the proteasome [19–23]. Interestingly, we also predicted that miR-183 may target VHL mRNA CDS using the RNA22 software (Figure 5A). Then, we cloned the MRE of VHL mRNA CDS and its mutant into pGL3-Control plasmid. Our data elucidated that miR-183 could remarkably decrease the luciferase activities of pLuc-VHL-397p-183 in MCF-7 cells. But, it failed to work when the miR-183-targeting sites of VHL mRNA CDS were mutated (Figure 5B). Meanwhile, anti-miR-183 was capable of increasing the luciferase activities of pLuc-VHL-397p-183 in MCF-7-HBXIP cells, which could be abolished when the target sites of miR-183 in VHL mRNA CDS were mutated (Figure 5C). Subsequently, we validated that miR-183 was able to downregulate VHL at the protein levels in MCF-7 cells, while anti-miR-183 led to the opposite results in MCF-7-HBXIP cells (Figure 5D and 5E). We are interested in whether miR-183 could enhance the stability of HIF1α in the event. As expected, miR-183 was able to increase the levels of HIF1α protein in MCF-7 cells (Figure 5F), but anti-miR-183 led to the opposite results in MCF-7-HBXIP cells (Figure 5G). Thus, we conclude that miR-183 enhances the stability of HIF1α through targeting VHL mRNA CDS in breast cancer cells.

### HBXIP enhances the glucose metabolism reprogramming of breast cancer cells through miR-183/96/182 *in vitro*

**Figure 6:**
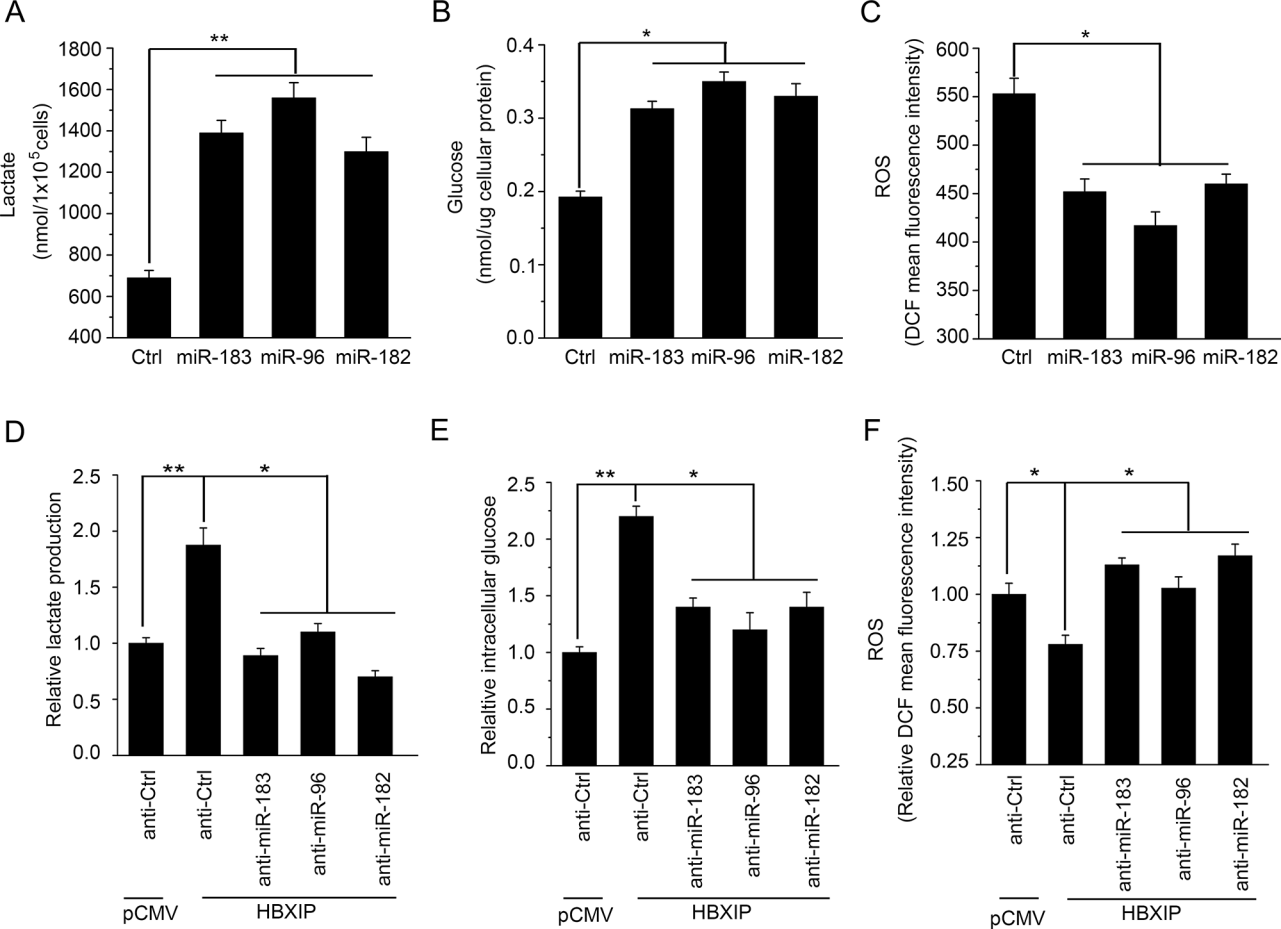
HBXIP enhances the glucose metabolism reprogramming of breast cancer cells through miR-183/96/182 *in vitro* **A.** The levels of lactate in the culture media of MCF-7 cells were measured by an Agilent 1100 series high-performance liquid chromatography (HPLC) system and normalized to cell number. **B.** The levels of intracellular glucose were detected by glucose-lactate biosense tester SBA-40E and normalized based on the protein concentration in MCF-7 cells. **C.** The levels of intracellular ROS were assessed by flow cytometry analysis in MCF-7 cells. **D–F.** The relative levels of lactate, intracellular glucose and ROS were measured using above methods in HBXIP-transfected MCF-7 cells treated with anti-miR-183 (anti-miR-96 or anti-miR-182). Statistically significant differences are indicated: *P < 0.05, **P < 0.01, Student's *t*-test. Each experiment was repeated at least three times. Data are shown as mean ± SEM (*n* = 3).

Based on the notion that miR-183/96/182 cluster inhibited the expression of SCO2 and PDHA1 through targeting SCO2 and PDHA1 mRNA CDSs, we further evaluated the effect of miR-183/96/182 on the glucose metabolism reprogramming in breast cancer cells. As expected, we found that all of them were able to significantly increase the levels of lactate production and intracellular glucose in MCF-7, T47D and 293T cells (Figure 6A and 6B; Supplementary Figure 4A and 4B). Moreover, the levels of ROS were significantly reduced in MCF-7, T47D and 293T cells (Figure 6C; Supplementary Figure 4C). To further validate the effect of HBXIP on the glucose metabolism reprogramming involving miR-183/96/182, we examined the levels of lactate production, intracellular glucose and ROS when the cells were treated with the inhibitors of miR-183, miR-96 or miR-182, respectively. Our data showed that the increases of lactate production and intracellular glucose mediated by HBXIP were significantly abrogated by the inhibitors of miR-183, miR-96 or miR-182 in MCF-7 cells, respectively (Figure 6D and 6E). But, the decreases of ROS mediated by HBXIP were rescued by these miRNA inhibitors (Figure 6F). Thus, we conclude that HBXIP enhances the glucose metabolism reprogramming of breast cancer cells through miR-183/96/182 *in vitro*.

### HBXIP enhances the growth of breast cancer cells through miR-183/96/182 targeting SCO2 and PDHA1 *in vivo*

**Figure 7:**
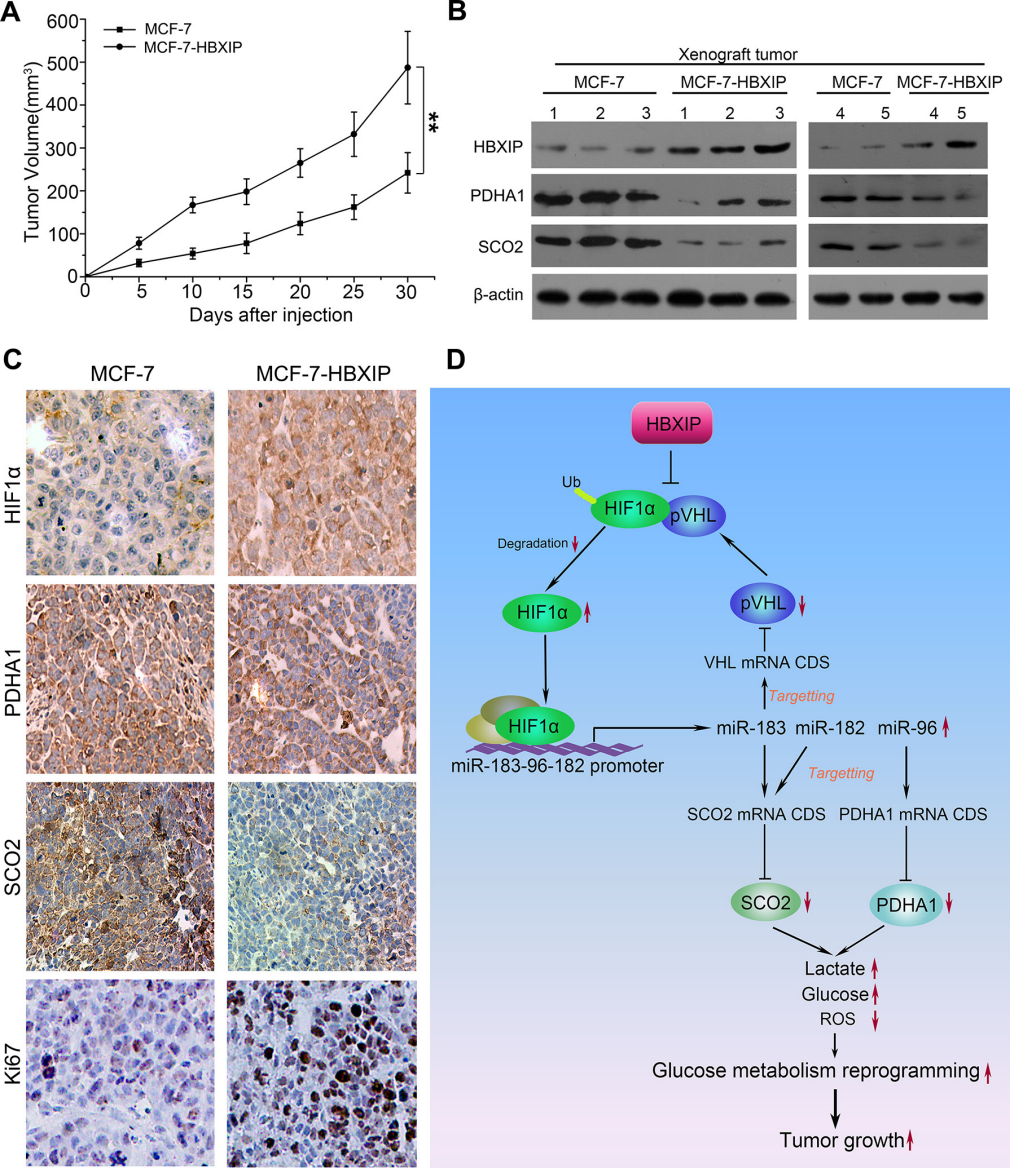
HBXIP enhances the growth of breast cancer cells through miR-183/96/182 targeting SCO2 and PDHA1 *in vivo* **A.** The growth curves of tumors derived from MCF-7 and MCF-HBXIP cells were shown. **B.** The protein levels of HBXIP, PDHA1, SCO2 and HIF1α were detected by Western blot analysis in the xenograft tumor tissues from above mice. **C.** The expression of PDHA1, SCO2, HIF1α and Ki67 was tested by IHC analysis in the tumor tissues from above mice. **D.** A model of HBXIP modulating SCO2 and PDHA1 involved in the glucose metabolism reprogramming is summarized, in which a feedback loop of HIF1α/miR-183/pVHL/HIF1α modulated by HBXIP contributes to the glucose metabolism reprogramming in breast cancer cells. Statistically significant differences are indicated: **P < 0.01, Student's *t*-test.

To investigate the effect of HBXIP on the glucose metabolism reprogramming of breast cancer cells *in vivo*, we performed the experiment of tumor xenograft in mice. Our data demonstrated that the tumor weight and volume of MCF-7-HBXIP group were significantly higher than those of MCF-7 group (Figure 7A; Supplementary Figure 5A and 5B). Furthermore, we validated the effect of HBXIP on PDHA1, SCO2 or HIF1α in xenograft tumor tissues by Western blot analysis and IHC analysis, respectively (Figure 7B and 7C). Compared with MCF-7 group, the expression of Ki67, a cell proliferation marker, was significantly increased in MCF-7-HBXIP group (Figure 7C). In addition, we showed that the expression levels of miR-183/96/182 were higher in the xenograft tumor tissues derived from MCF-7-HBXIP cells than those from MCF-7 cells (Supplementary Figure 5C). Thus, we conclude that HBXIP promotes the growth of breast cancer cells through miR-183/96/182 targeting SCO2 and PDHA1 *in vivo*.

## DISCUSSION

The altered metabolism in cancer cells confers a selective advantage for their survival and proliferation through facilitating the uptake and incorporation of nutrients into the biomass (e.g., nucleotides, amino acids and lipids) and minimizing the production of reactive oxygen species in mitochondria [1, 3]. It has been reported that the metabolic switch may provide a benefit to cancers not only by increasing glycolysis but also by decreasing mitochondrial activity [17]. The “Warburg effect” is one of the key hallmarks of cancer cells. The glucose metabolism reprogramming contributes to the development of cancer. However, the mechanism remains poorly defined. Accumulated evidence has reported that the oncoprotein HBXIP plays crucial roles in the development of breast cancer [28–33]. In this study, we are interested in whether HBXIP is involved in the glucose metabolism reprogramming in breast cancer.

Interestingly, we found that HBXIP was involved in the regulation of the glucose metabolism reprogramming in breast cancer cells. SCO2 and PDHA1 are closely associated with the metabolic switch from OXPHOS to glycolysis [6, 7]. Accordingly, we validated that HBXIP was able to downregulate the expression levels of SCO2 and PDHA1 in breast cancer cells. Moreover, we predicted that miR-183 and miR-182 might target SCO2 mRNA CDS, and miR-96 might target PDHA1 mRNA CDS. It has been reported that the miR-183/96/182 cluster is an evolutionarily conserved miRNA cluster, and their altered expressions are involved in the initiation and progression of human cancers [35–37, 41]. Our data showed that miR-183/96/182 cluster was able to downregulate the expression of SCO2 and PDHA1 in breast cancer cells at the post-transcriptional level. It suggests that the miR-183/96/182 cluster might be involved in the glucose metabolism reprogramming through targeting SCO2 and PDHA1 in breast cancer cells.

Moreover, we identified that HBXIP was able to upregulate miR-183/96/182 through activating its promoter involving transcriptional factor HIF1α in breast cancer cells. It has been reported that the transcriptional factor HIF1 complex, consisting of the regulated HIF1α and the constitutively expressed HIF1β, increases virtually all the enzymes in the glycolytic pathway as well as the glucose transporters 1 and 3 (GLU1, GLU3) [42, 43]. Targeting aerobic glycolysis and HIF1α expression enhance drug-induced apoptosis in cancer cells [44]

HIF1α is unstable in well-oxygenated tissues owing to ubiquitin-mediated degradation. The main action of pVHL is thought to be its E3 ubiquitin ligase activity that results in degradation of specific target proteins, in which HIF1α is one of the most researched targets [19, 21]. Previously, we reported that HBXIP was able to upregulate HIF1α expression through FGF8/PI3K/Akt signaling in MCF-7 cells [33]. In this study, we found that HBXIP could increase the protein level of HIF1α through inhibition of ubiquitin-mediated degradation of HIF1α as well. Furthermore, we identified that HBXIP disassociated the interaction of HIF1α with pVHL in breast cancer cells. It is consistent with the previous report that the forced expression of ubiquitin carrier protein (UCP) caused the proteasomal-dependent degradation of pVHL, resulting in the accumulation of HIF1α in normoxia [18]. The modulation of network in cancer cells is frequently occurred in a positive feedback loop manner [45, 46]. Interestingly, in this study we found that the forced expression of miR-183 could significantly increase HIF1α expression through targeting VHL mRNA CDS. Therefore, a feedback loop of HIF1α/miR-183/pVHL/HIF1α modulated by HBXIP contributes to the glucose metabolism reprogramming in breast cancer cells. In function, we validated that HBXIP was able to accelerate the glucose metabolism reprogramming through upregulating miR-183/96/182 cluster *in vitro* and *in vivo*. Therapeutically, HBXIP may acts as a new target for breast cancer.

Taken together, we summarize a model of HBXIP modulating SCO2 and PDHA1 involved in the glucose metabolism reprogramming in Figure 7D. HBXIP disassociates the interaction of HIF1α with pVHL, resulting in the increase of stability of HIF1α in breast cancer. Furthermore, HBXIP activates the promoter of miR-183/96/182 cluster through transcriptional factor HIF1α. MiR-183 and miR-182 can target the CDSs of SCO2 and PDHA1 mRNAs and miR-96 can target the CDS of pVHL mRNA in the cells. The downregulation of pVHL results in the increase of stability of HIF1α. The downregulation of SCO2 and PDHA1 leads to the increase of lactate production and intracellular glucose, and the decrease of ROS, promoting the growth of breast cancer cells. Therefore, a feedback loop of HIF1α/miR-183/pVHL/HIF1α modulated by HBXIP contributes to the glucose metabolism reprogramming in breast cancer cells. Thus, our finding provides new insights into the mechanism of the glucose metabolism reprogramming mediated by HBXIP in breast cancer.

## MATERIALs AND METHODS

### Cell lines

MCF-7, T47D and MCF-7-HBXIP (MCF-7 stably transfected with HBXIP) cells were cultured in RPMI-1640 media (Gibco, CA, USA) with 10% fetal bovine serum (FBS). HEK293T cells were cultured in Dulbecco's modified Eagle's medium (Gibco) with 10% FBS.

### Patient samples

The patient samples used in this study included breast cancer tissue microarrays containing 95 breast cancer tissue samples for immunohistochemistry analysis, 30 pairs of tumorous and adjacent non-tumorous breast samples for quantitative real-time PCR analysis and 8 selected from 30 breast cancer tissues for immunohistochemistry analysis. The breast cancer tissues and their adjacent non-tumorous tissues were collected from patients undergoing resection for breast cancer in Tumor Hospital of Tianjin Medical University. Informed consent was obtained from each patient and the study was approved by the Institutional Research Ethics Committee in Nankai University.

### Plasmids and oligonucleotides

The coding sequence (CDS) region of human SCO2 or PDHA1 was amplified by PCR using the cDNA from MCF-7 cells as template, which was cloned into pCMV-tag2B vector, respectively. The CDS region of HIF1α or a prolyl-hydroxylation-defective mutant of HIF1α (P564A) was amplified by PCR using the cDNA from MCF-7 cells as template, which was cloned into pEGFP-C2 vector. MiR-183/96/182 mimics (miR-183/96/182), negative control (Ctrl), miR-183/96/182 inhibitor (anti-miR-183/96/182) and inhibitor negative control (anti-Ctrl) were synthesized by RiboBio (Guangzhou, China). All oligonucleotide sequences are listed in Supplementary Table 1.

### Cellular glucose detection

Transfected MCF-7 and T47D cells were collected and washed three times with phosphate-buffered saline (PBS). The lysate was centrifuged at 14,000 g for 15 min at 4°C. The supernatant was used for measurement of glucose by glucose-lactate biosense tester SBA-40E (Institute of Biology, Shandong Academy of Sciences, Jinan, China).

### Lactate production detection

Transfected MCF-7 and T47D cells were cultured for 24 h. The lactate levels in the culture media were determined by an Agilent 1100 series high-performance liquid chromatography (HPLC) system (Hewlett-Packard Corporation, USA) [47].

### ROS detection

Transfected MCF-7 and T47D cells were cultured for 24 h, and then the cells were incubated with FBS-free culture medium containing 10 μM DCFH-DA for 20 min at 37°C. DCFH-DA was metabolized by the non-specific esterase to the non-fluorescence product, DCFH, which was oxidized to the fluorescent product, DCF, by ROS. The cells were washed three times with PBS to remove the unabsorbed DCFH-DA and then trypsinized, re-suspended in PBS, and measured for their ROS using flow cytometer.

### Vector construction

RNA22 was used to predict miRNA targets (miRNA response elements, MREs). According to the report [34], we have denoted these sites as X-Yp-Z, in which X is the targeted gene, Y is the distance (in bases) of the 5′ end of the predicted target from the translation initiation site, Z represents the targeting miRNA (miR-183, miR-96 or miR-182), and p stands for ‘prediction’. For pLuc-MRE constructs, the sequences containing the RNA22-predicted miRNA-MREs were subcloned into the XbaI/FseI site, the downstream of the luciferase gene in the pGL3-Control vector, to generate pLuc-SCO2-663p-183-wt, pLuc-SCO2-96p-182-wt, pLuc-PDHA1-419p-96-wt and pLuc-VHL-397p-183-wt. Mutant constructs of MREs were termed pLuc-SCO2-663p-183-mut, pLuc-SCO2-96p-182-mut, pLuc-PDHA1-419p-96-mut and pLuc-VHL-397p-183-mut. The 5′-flanking different fragments of miR-183/96/182 locus promoter, including −7830~ −5573 (P1), −7830~ −6487 (P2), −6987~ −5573 (P3), −6987~ −6487(P4), −7830~ −6957 (P5) and −6507~ −5573 (P6), were amplified by PCR using the genomic DNA from MCF-7 cells as template, which were cloned into the upstream of the pGL3-Basic vector (Promega, Madison, WI USA). All primers are listed in Supplementary Table 1.

### Luciferase reporter gene assays

MiRNA target validation assays were performed as described previously [48]. MCF-7 or HEK293T cells were seeded in 24-well plates for 12 h before transfection. MiRNA mimics or the negative control were co-transfected at a final concentration of 75 nM with 100 ng of pLuc-MRE. MCF-7 cells were treated with a chemical inducer of HIF1α, cobalt chloride (CoCl_2_, Sigma-Aldrich, St Louis, MO, USA), at a final concentration of 500 μM. In all cases, pRL-TK, a constitutively expressed Renilla luciferase plasmid, was used as a normalization control for transfection efficiency. Thirty-six hours after transfection, firefly and Renilla luciferase activities were measured consecutively with the dual-luciferase reporter system (Promega, Madison, WI, USA).

### Western blot analysis, reverse transcription—polymerase chain reaction (RT-PCR), quantitative real-time PCR (qRT-PCR) and immunohistochemistry analysis (IHC)

These experiments of Western blot, RT-PCR, qRT–PCR and IHC were carried out as described previously [28, 29]. For Western blot analysis, the primary antibodies were mouse anti-HBXIP monoclonal antibody (Abcam, Cambridge, UK), rabbit anti-p53 polyclonal antibody (Proteintech Group, Chicago, IL, USA), rabbit anti-SCO2 polyclonal antibody (Proteintech Group), rabbit anti-PDHA1 polyclonal antibody (Proteintech Group), rabbit anti-HIF1α polyclonal antibody (Proteintech Group), rabbit anti-pVHL polyclonal antibody (Proteintech Group), rabbit anti-ubiquitin polyclonal antibody (Proteintech Group) and mouse anti-β-actin monoclonal antibody (Sigma-Aldrich). QRT-PCR was exploited to detect the expression levels of miRNAs and genes using Absolute Blue qRT-PCR SYBR green mix (TaKaRa Bio, Dalian, China) according to the manufacturer's instructions. Double-stranded DNA specific expression was examined by the comparative Ct method using 2^−ΔΔCt^. Primers were listed in Supplementary Table S1. For IHC analysis, breast cancer tissue microarrays (NO. C0048) obtained from Xi'an Aomei Biotechnology Co., Ltd (Xi'an, China) were stained using antibodies against HBXIP (Abcam), SCO2 (Proteintech Group) and PDHA1 (Proteintech Group). The percentage of immunoreactivity in breast cancer tissues or normal breast tissues was graded as: 0 (−), (<10%); 1 (+), low (10% – 30%); 2 (++), intermediate (30%–50%); 3 (+++), high (>50%). Categorization of immunostaining intensity was performed by three independent observers.

### Chromatin immunoprecipitation (ChIP), co-immunoprecipitation (co-IP) and electrophoretic mobility shift assays (EMSA)

These experiments were described previously [28, 33]. For ChIP assay, protein-DNA complexes were immunoprecipitated with anti-HIF1α antibody. Normal IgG was used as a negative control. For co-IP assay, the cell lysates and protein G beads with respective primary antibodies were mixed and incubated overnight at 4°C. After eight rounds of washes with the same lysis buffer, co-precipitated proteins were eluted, and then detected by Western blot analysis. For EMSA, nuclear extracts from MCF-7 were prepared. Oligonucleotides of miR-183/96/182 promoter were labeled with ^32^P-γATP using T4 polynucleotide kinase (TaKaRa Bio).

### Tumor xenograft in mouse assays

Four-week-old BALB/c athymic nude mice were purchased from the Experiment Animal Center of Beijing (Beijing, China) for study approved by the National Institutes of Health Guide for the Care and Use of Laboratory Animals. The mice (each group, *n* = 5) were subcutaneously injected with (4 × 10^5^) MCF-7 or MCF-7-HBXIP cells. After 4 w, the animals were killed to remove tumors for analysis.

### Statistical analysis

Differences were considered to be statistically significant by Student's *t*-test, *P*-value < 0.05. The expression levels of miRNAs in breast cancer tissues and their corresponding nontumorous tissues were analyzed using Wilcoxon signed-rank test. Pearson's correlation coefficient was used to determine the correlation between the miRNA levels and the gene expression in clinical breast cancer tissues. Each experiment was repeated at least three times.

## SUPPLEMENTARY FIGURES AND TABLE


