# Supplementary material for: The oncoprotein HBXIP promotes glucose metabolism reprogramming via downregulating SCO2 and PDHA1 in breast cancer
Source: Oncotarget. 2015 Jul 30;6(29):27199–213. doi: 10.18632/oncotarget.4508 (PMC4694983; doi:10.18632/oncotarget.4508)
Supplement: Supplementary file 1 [file oncotarget-06-27199-s001.pdf]

SUPPLEMENTARY FIGURES AND TABLE

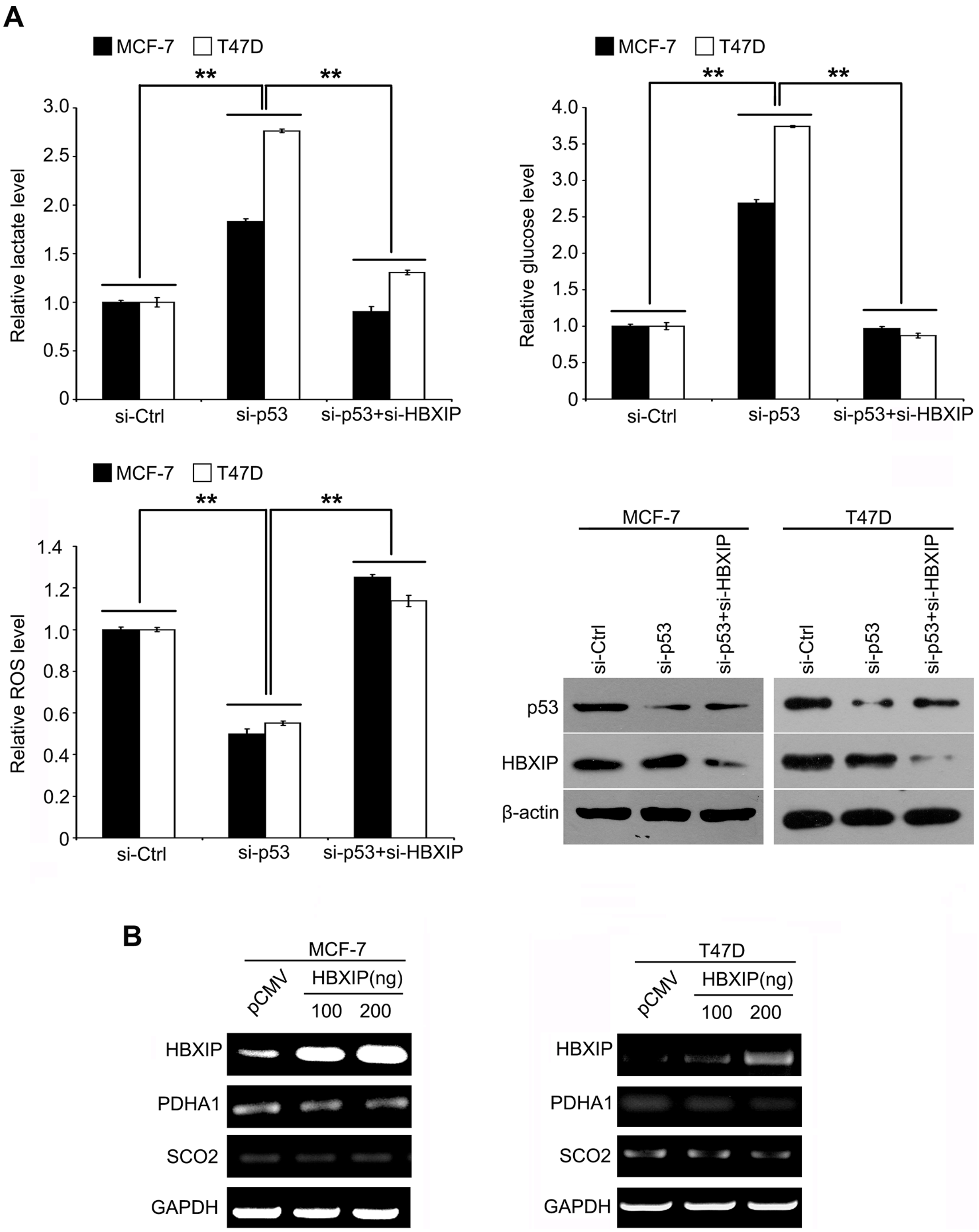

Supplementary Figure 1: HBXIP downregulates the expression of SCO2 and PDHA1 in breast cancer cells. A. The levels of lactate, intracellular glucose and ROS were measured by HPLC, glucose-lactate biosense tester SBA-40E and flow cytometer analysis in MCF-7 and T47D cells transfected with si-p53 or si-p53/si-HBXIP. The interference efficiency of si-p53 and si-HBXIP was confirmed by Western blot analysis. **B.** The mRNA levels of HBXIP, SCO2 and PDHA1 were examined by RT-PCR analysis in MCF-7 and T47D cells transfected with HBXIP. Statistically significant differences are indicated: $^{**}P < 0.01$, Student's *t*-test. Each experiment was repeated at least three times. Data are shown as mean ± SEM ($n = 3$).

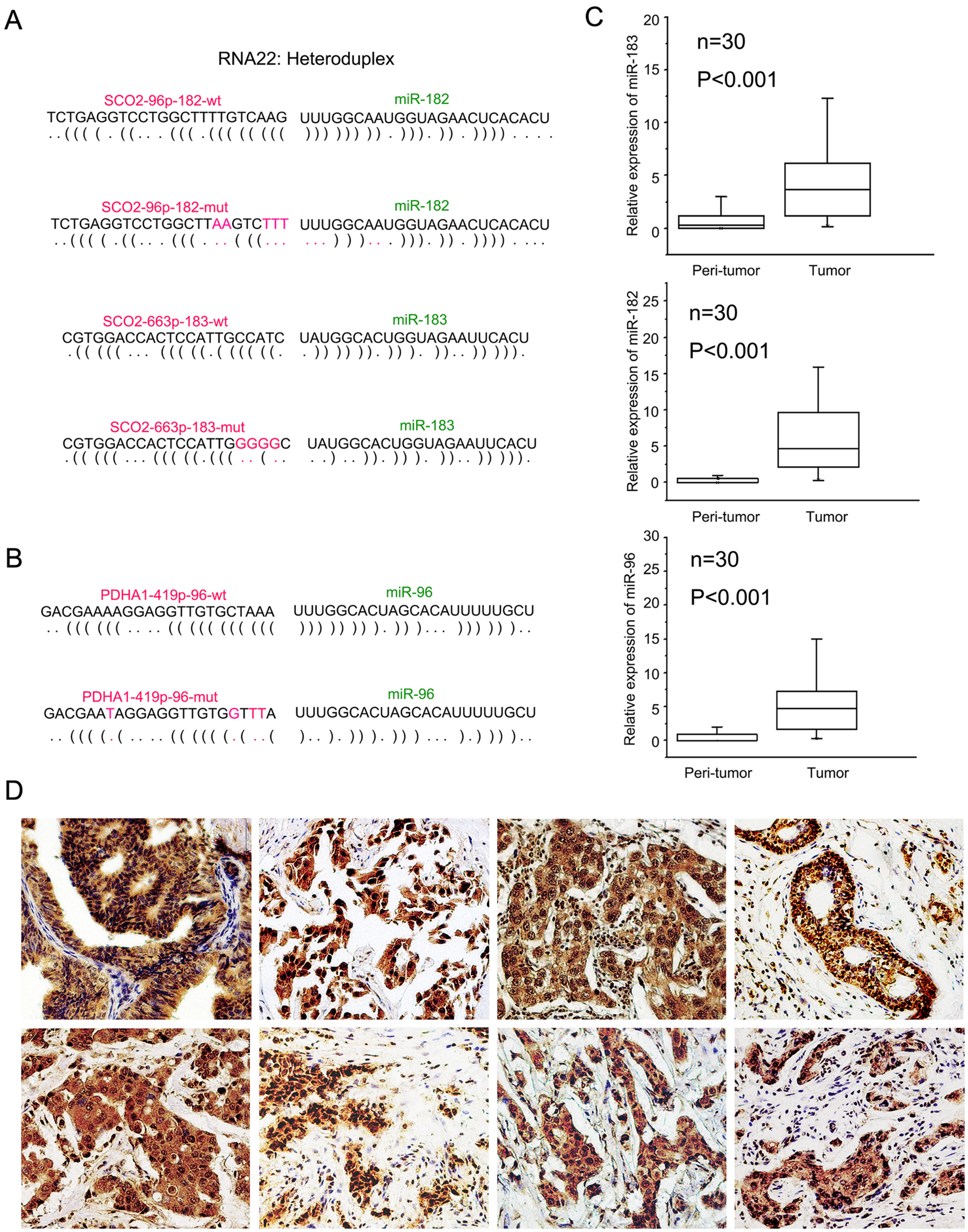

Supplementary Figure 2: MiR-183/96/182 cluster targets SCO2 and PDHA1 mRNA CDSs. A, B. The schematic diagram shows the heteroduplexes of the mRNA CDSs of SCO2 and PDHA1 and their mutants in the miRNA recognizing elements (MREs) of miR-183, miR-182 and miR-96. **C.** The relative expression levels of miR-183, miR-182 and miR-96 were detected by qRT-PCR analysis in 30 pairs of clinical breast cancer tissues and their surrounding non-tumorous tissues ($P < 0.001$, Wilcoxon signed-rank test). **D.** The HBXIP expression was examined by IHC analysis in 8 selected from 30 clinical breast cancer tissues samples. Each experiment was repeated at least three times. Data are shown as mean ± SEM ($n = 3$).

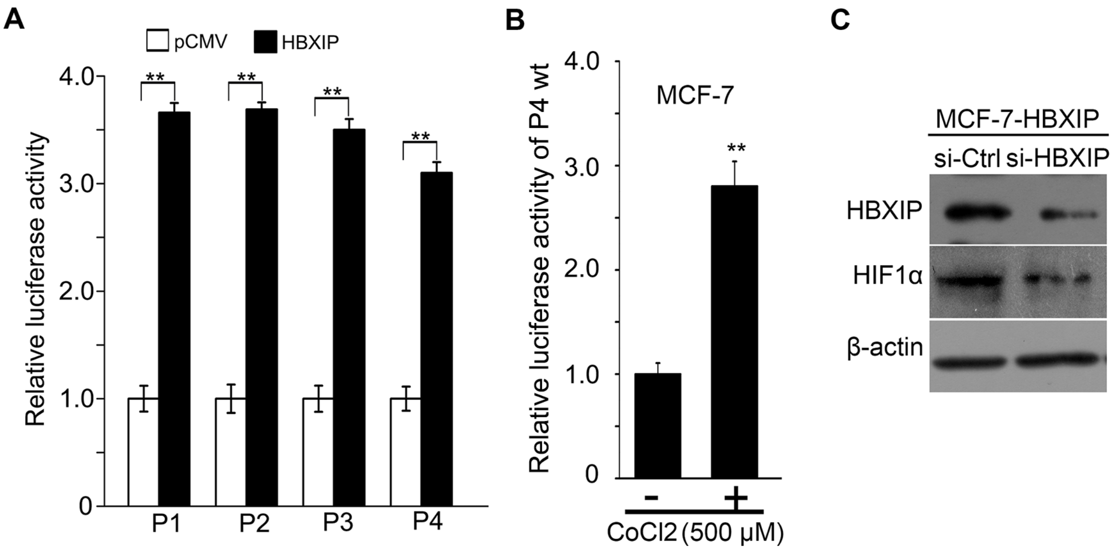

Supplementary Figure 3: HBXIP activates miR-183/96/182 promoter through transcriptional factor HIF1α. A. The promoter activities of miR-183/96/182 cluster were analyzed by luciferase reporter gene assays in MCF-7 cells transfected with HBXIP. **B.** The effect of CoCl2 (500 μM), a chemical inducer of HIF1α, on the promoter activity of P4 fragment of miR-183/96/182 was detected by luciferase reporter gene assays in MCF-7 cells. **C.** The protein levels of HIF1α and HBXIP were detected by Western blot analysis in MCF-7-HBXIP cells transfected with si-HBXIP. Statistically significant differences are indicated: $^{**}P < 0.01$, Student's *t*-test. Each experiment was repeated at least three times. Data are shown as mean ± SEM ($n = 3$).

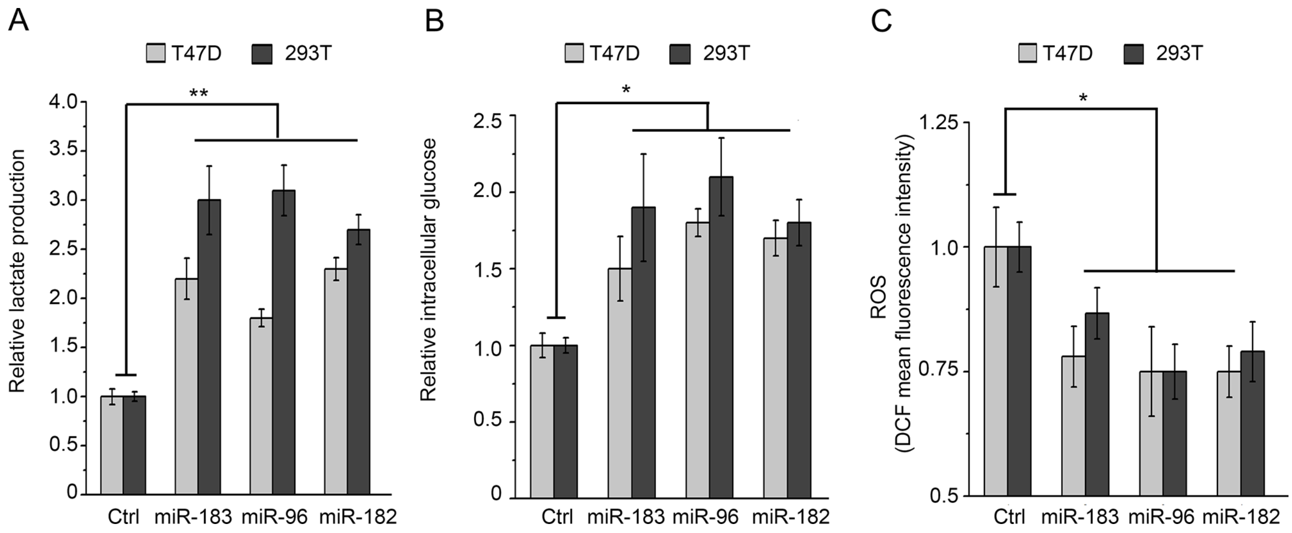

Supplementary Figure 4: HBXIP enhances the glucose metabolism reprogramming of breast cancer cells through miR-183/96/182 *in vitro*. A. The levels of lactate in the culture media of T47D and 293T cells transfected with miR-183/96/182 were measured by HPLC and normalized to cell number. **B.** The levels of intracellular glucose were detected by glucose-lactate biosense tester SBA-40E and normalized based on the protein concentration in T47D and 293T cells transfected with miR-183/96/182. **C.** The levels of intracellular ROS were assessed by flow cytometry in T47D and 293T cells transfected with miR-183/96/182. Statistically significant differences are indicated: $^{*}P < 0.05$, $^{**}P < 0.01$, Student's *t*-test. Each experiment was repeated at least three times. Data are shown as mean ± SEM ($n = 3$).

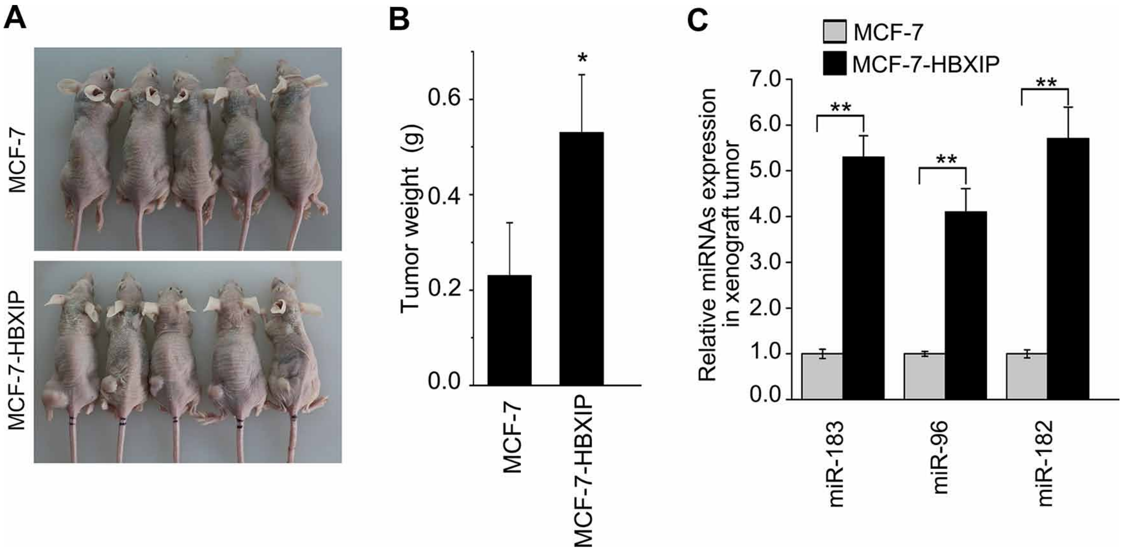

Supplementary Figure 5: HBXIP enhances the growth of breast cancer cells through miR-183/96/182 targeting SCO2 and PDHA1 *in vivo*. A. Photographs of MCF-7 cells and MCF-7-HBXIP cells-injected nude mice with tumors were shown. **B.** The average weight of tumors from above mice was shown. **C.** The relative expression levels of miR-183, miR-96 and miR-182 were detected by qRT-PCR analysis in the xenograft tumor tissues derived from MCF-7 and MCF-7-HBXIP cells. Statistically significant differences are indicated: $^{*}P < 0.05$, $^{**}P < 0.01$, Student's *t*-test. Each experiment was repeated at least three times. Data are shown as mean ± SEM ($n = 3$).

Supplementary Table 1: List of primers used in this paper

Gene	Primer	Sequence (5′-3′)
**Primers for miR-183/96/182 promoter**		
P1	forward	CTTGTATCCCGGGCACCTTT
	reverse	TGCTTGGAAATCCGACCCTG
P2	forward	CTTGTATCCCGGGCACCTTT
	reverse	TTCAATATGCCCGACTCCCG
P3	forward	GGTTCTCGACAGCAGGTGAA
	reverse	TGCTTGGAAATCCGACCCTG
P4	forward	GGTTCTCGACAGCAGGTGAA
	reverse	TTCAATATGCCCGACTCCCG
P5	forward	CTTGTATCCCGGGCACCTTT
	reverse	TTCACCTGCTGTCGAGAACC
P6	forward	CGGGAGTCGGGCATATTGAA
	reverse	TGCTTGGAAATCCGACCCTG
P4-hif1α-mut	forward	GGCCCGTTGGGCTCTGTGACTCATTATCTCCGCTGTCCGGGTTCTCGCT
	reverse	AGCGAGAACCCGGACAGCGGAGATAATGAGTCACAGAGCCCAACGGGCC
**miRNAs qRT-PCR primers**		
miR-183	forward	TATGGCACTGGTAGAATTCACT
miR-96	forward	TTTGGCACTAGCACATTTTTGCT
miR-182	forward	TTTGGCAATGGTAGAACTCACACT
	reverse	TAGCAGCGGGAACAGTTCTGCAG

(Continued)

Gene	Primer	Sequence (5′-3′)
**Primers for RT-PCR and qRT-PCR**		
PDHA1	forward	AGACCATCTCATCACAGCCTAC
	reverse	TCTCAACAGACGTTCCCATT
SCO2	forward	TGGGTGCTGATGTACTTTGGC
	reverse	ACAGTCTTGGGTGGAAGTCCTG’
GAPDH	forward	CATCACCATCTTCCAGGAGCG
	reverse	TGACCTTGCCCACAGCCTTG
HBXIP	forward	ATGGAGCCAGGTGCAGGTC
	reverse	TGGAGGGATTCTTCATTGTG
**Primers for CDS cloning**		
SCO2	forward	ATGCTGCTGCTGACTCGGAGCC
	reverse	TCAAGACAGGACACTGCGGAAA
PDHA1	forward	ATGAGGAAGATGCTCGCCG
	reverse	TTAACTGACTGACTTAAACTTG
HIF1α	forward	CGGGGTACCATGGAGGGCGCCGGCGGC
	reverse	CGCGGATCCTCAGTTAACTTGATCCAAAGCTCTG
HIF1(P564A)	forward	ATTTAGACTTGGAGATGTTAGCTGCCTATATCCCAATGGATGATGACTT
	reverse	AAGTCATCATCCATTGGGATATAGGCAGCTAACATCTCCAAGTCTAAAT
**Primers for ChIP**		
	forward	ATGGGTCTGAGGACCAGGTAGG
	reverse	CCCAGGTGGATAACTGGACGA
**Primers for pLuc-SCO2-96p-182**		
	forward	TCAGCTCAAGCCTCGGGTCC
	reverse	TCCAGCCCCGAACAGGC
**Primers for pLuc-SCO2-663p-183**		
	forward	TGACATCTGCCCAGACGAGC
	reverse	GACAGGACACTGCGGAAAGC
**Primers for pLuc-PDHA1-419p-96**		
	forward	ACAGACCATCTCATCACAGCCTAC
	reverse	TAGCAGCACCATCGCCATAT
Si-HBXIP	GCAGCUAAGCUAACCUCUGdTdT	
Si-p53	GUGCAGCUGUGGGUUGAUUUU	
